# HSV1 microRNAs in glioblastoma development: an in silico study

**DOI:** 10.1038/s41598-023-45249-2

**Published:** 2024-01-02

**Authors:** Mahdi Abdoli Shadbad, Nima Hemmat, Mahla Abdoli Shadbad, Oronzo Brunetti, Nicola Silvestris, Behzad Baradaran

**Affiliations:** 1 Student Research Committee, Tabriz University of Medical Sciences, Tabriz, Iran; 2 Immunology Research Center, Tabriz University of Medical Sciences, Tabriz, Iran; 3 European Virus Bioinformatics Center (EVBC), 07743 Jena, Germany; 4 Medical Oncology Unit-IRCCS Istituto Tumori “Giovanni Paolo II” of Bari, Bari, Italy; 5 Medical Oncology Unit, Department of Human Pathology “G. Barresi”, University of Messina, Messina, Italy

**Keywords:** CNS cancer, Microarrays

## Abstract

Glioblastoma multiforme (GBM) is a highly aggressive primary brain tumor. Recent findings highlighted the significance of viral microRNAs (miRs) in regulating post-transcriptional mRNA expression in various human conditions. Although HSV1 encodes viral miRs and affects the central nervous system, no study investigated the roles of HSV1-encoding miRs in GBM development. This study applied in silico approaches to investigate whether HSV1-encoding miRs are involved in GBM development and, if so, how they regulate tumor-suppressive/oncogenes expression in GBM. This study leveraged bioinformatics approaches to identify the potential effect of HSV1 miRs in GBM development. The GSE158284, GSE153679, and GSE182109 datasets were analyzed to identify differentially expressed genes in GBM tissues and cell lines using the *limma* package in the R software. The GSE182109 dataset was analyzed to determine gene expression at the single-cell levels using the *Seurat* package in the R software. The TCGA-GTEX, GDSC, CTRP, immunogenetic, and enrichment analyses were performed to study the impact of identified viral HSV1 miRs targets in GBM development. hsv1-miR-H6-3p is upregulated in GBM and can be responsible for *EPB41L1* and *SH3PXD2A* downregulation in GBM tissues. Also, hsv1-miR-H1-5p is upregulated in GBM and can decrease the expression of *MELK*, *FZD2*, *NOVA1*, *TMEM97*, *PTPRZ1*, and *PDGFC* in GBM development. The single-cell RNA sequencing analyses have demonstrated that *MELK*, *FZD2*, *NOVA1*, *TMEM97*, *PTPRZ1*, and *PDGFC* are expressed in astrocytes residing in the GBM microenvironment. This study provides novel insights into the potential roles of HSV1 miRs in GBM pathogenesis and offers a reference for further studies on the significance of HSV1 miRs in GBM development.

## Introduction

Glioblastoma multiforme (GBM) is a commonly diagnosed primary brain malignancy. In the United States, GBM is the most frequently diagnosed central nervous system tumor in adults. However, unremarkable improvement has been made in increasing the 5-year GBM patients' survival^1^. Understanding GBM biology can pave the way for introducing new treatments for affected patients.

Growing evidence has highlighted the significance of microRNA (miR) in developing various malignancies; miRs are non-protein-coding RNAs that can post-transcriptionally regulate various mRNAs^2–4^. It is well-established that miRs have pivotal roles in different biological processes^5^. In cancer biology, specific miRs can be divided into tumor-suppressive miRs and oncomiRs, which their ectopic expression causes tumor suppression or tumor growth, respectively^6^. Therefore, understanding the biology of miRs in cancer development might provide valuable insights into cancer treatment.

Most human herpesviruses can encode miRs, and viral miRs have considerable roles in various pathogenesis and virus-related diseases^7^. For instance, it is well-known that EBV is associated with solid and hematological malignancies. Lei et al. have shown that ectopic expression of ebv-miR-BART3* downregulates the expression of *DICE1*, a tumor-suppressive gene, leading to increased cell proliferation in nasopharyngeal carcinoma^8^. ebv-miR-BART18-5p is substantially upregulated in nasopharyngeal carcinoma tissues, and its expression level is negatively correlated with *PTEN*, as a critical tumor-suppressive gene of the AKT/PI3K signaling pathway^9^. Consistent with these, HSV1 antibody positivity combined with other risk factors is associated with an increased risk of oropharyngeal squamous cell carcinoma development^10^. Based on a report, 15% of sequenced oropharyngeal squamous cell carcinoma tissues are positive for HSV^11^. Growing reports have shown the presence of HSV1 infection in malignant tissues, like vulvar and buccal squamous cell carcinoma^12,13^. Although HSV1 can infect the central nervous system, there is no comprehensive study to investigate the potential role of HSV1 in the development of GBM.

In the current study, we aimed to investigate whether HSV1 miRs are involved in GBM pathogenesis and, if they are involved, how they exert their potential oncogenic or tumor-suppressive effect on GBM development. For this aim, we conducted a deep in silico investigation, did single-cell RNA sequencing analyses, and performed a thorough literature review to identify the potential trace of HSV1 miRs in GBM development. We also used the TCGA-GBM dataset to externally validate the identified differentially expressed genes. The results of this study provide novel and valuable insights into the potential roles of HSV1 in GBM development.

## Results

### Sample normalization and clustering

**Figure 1:**
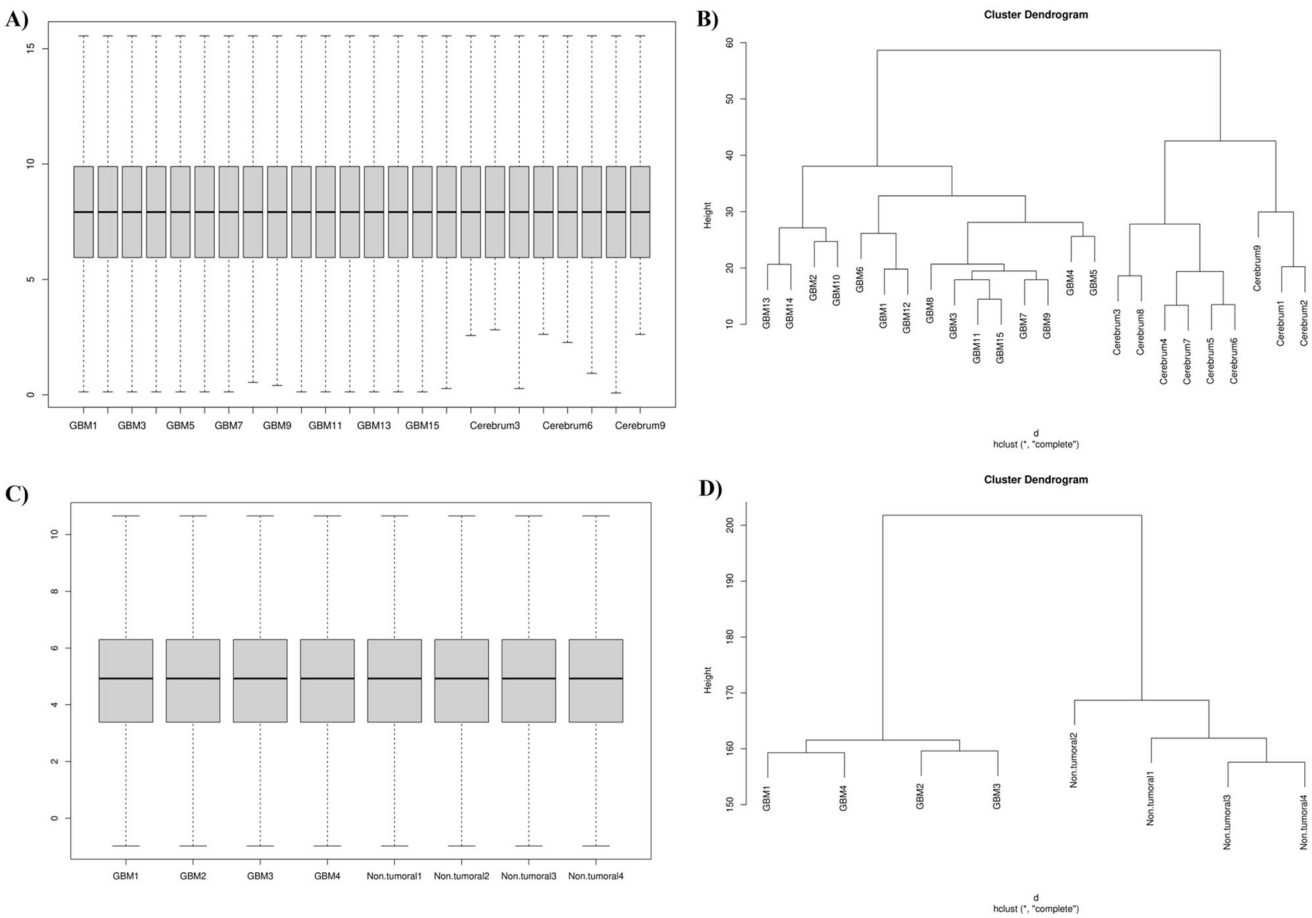
Data normalization and clustering. (**A**) The boxplot of normalized data of the included samples from the GSE158284 dataset. (**B**) Clustering of the included samples from the GSE158284 dataset. (**C**) The boxplot of normalized data of the included samples from the GSE13276 dataset. (**D**) Clustering of the included samples from the GSE13276 dataset.

First, we normalized the expression value of both the GSE158284 and GSE13276 datasets. After excluding 17 samples (nine samples were from pediatric GBM patients, six samples were not arranged based on unsupervised clustering, and two samples had technical problems in sequencing), we included 24 samples, i.e., 15 adult GBM tissues and 9 non-tumoral samples, from the GSE158284. The box plot of normalized values and clustering analysis of the 24 samples from the GSE158284 dataset are shown in Fig. 1A,B. Regarding the GSE13276 dataset, we included 8 samples from adult patients with GBM. We excluded 7 samples because they were disarranged according to unsupervised clustering or the non-tumoral tissues were not from adjacent GBM. The box plot of normalized values and clustering analysis of the 8 samples from the GSE13276 dataset are shown in Fig. 1C,D.

### Identifying differentially expressed genes and HSV1 miRs and their targets

**Figure 2:**
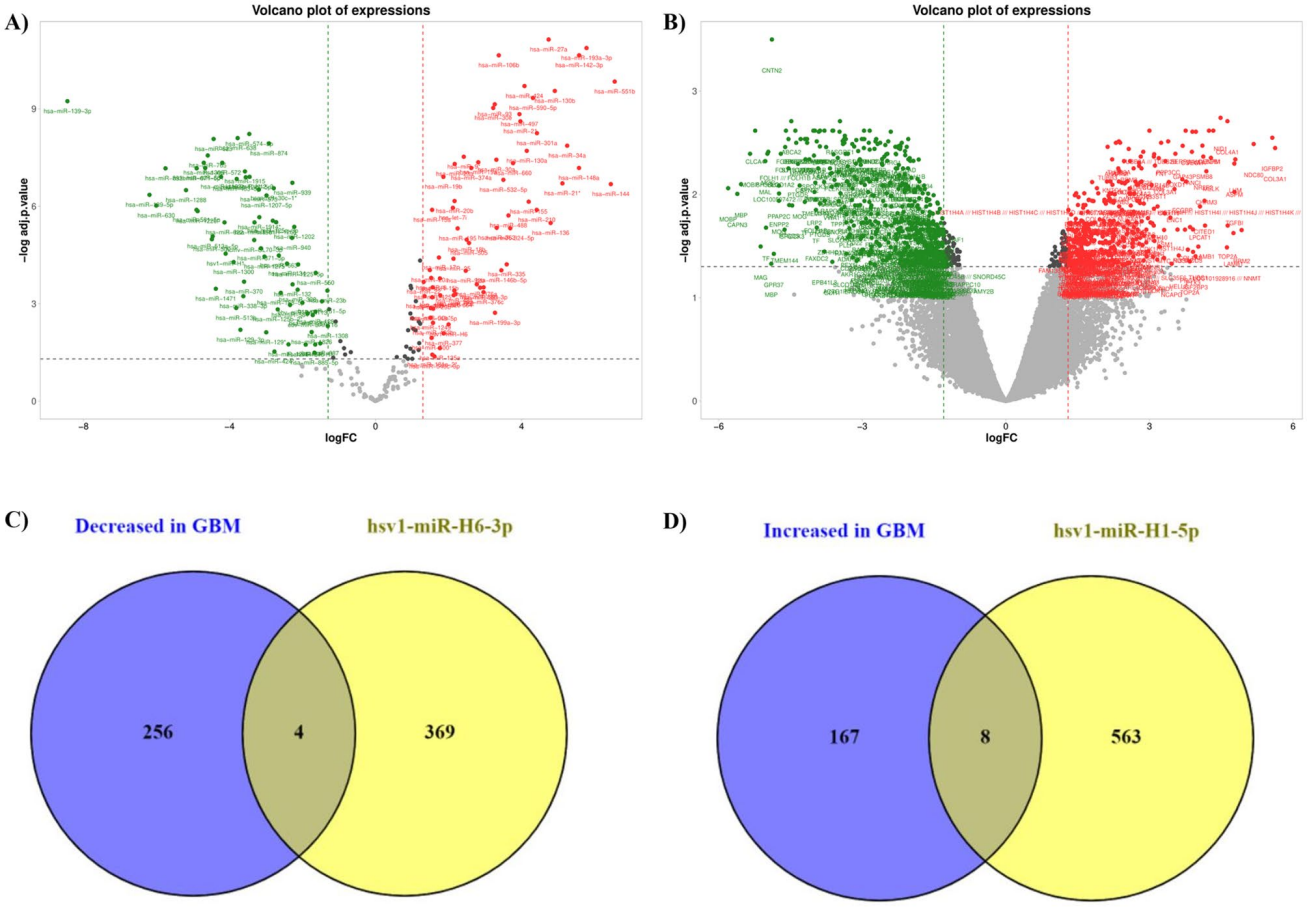
The volcano plots of the included samples from the GSE158284 and GSE13276 datasets and the common genes between DEGs and targets of hsv1-miR-H6-3p and hsv1-miR-H1-5p. (**A**) The differentially expressed genes (DEGs) of included samples from the GSE158284 dataset with the cut-off of adjusted P-value ˂ 0.05 and |log2FC| ≥ 1.3. (**B**) The DEGs of the included samples from the GSE158284 dataset adjusted P-value ˂ 0.05 and |log2FC| ≥ 1.3. (C) The common genes between DEGs and targets of hsv1-miR-H6-3p and hsv1-miR-H1-5p. (**C**) The common genes between targets of hsv1-miR-H6-3p and the downregulated genes in GBM tissues. (**D**) The common genes between targets of hsv1-miR-H1-5p and the upregulated genes in GBM tissues.

Afterward, we used the *limma* package to identify differentially expressed HSV miRs and differentially expressed genes in GBM tissues. By defining the adjusted P-value ˂ 0.05 and |log2FC| ≥ 1.3, we depicted the differentially expressed miRs and differentially expressed genes in GBM tissues (Fig. 2A,B, respectively). Regarding the HSV miRs, our results have indicated that hsv1-miR-H6-3p is significantly upregulated in adult GBM tissues, and hsv1-miR-H1-5p is significantly downregulated in adult GBM tissues. Afterward, we used the miRDB database to predict the potential targets of hsv1-miR-H6-3p and hsv1-miR-H1-5p. It is found that hsv1-miR-H6-3p can target 371 human mRNAs, and hsv1-miR-H1-5p can target 571 human mRNAs. Our results have indicated that four targets of hsv1-miR-H6-3p, as the upregulated HSV1 miR in GBM tissues, are among the significantly downregulated genes in GBM tissues; these genes are *SH3TC2*, *SASH1*, *EPB41L1*, and *SH3PXD2A* (Fig. 2C and Table 1). Also, eight targets of hsv1-miR-H1-5p, as the downregulated HSV1 miR in GBM tissues, are among the significantly upregulated genes in GBM tissues; these genes are *MELK*, *FZD2*, *IL1RN*, *NOVA1*, *MYH10*, *TMEM97*, *PTPRZ1*, and *PDGFC* (Fig. 2D, and Table 1).

**Table 1:** The differentially expressed targets of hsv1-miR-H6-3p and hsv1-miR-H1-5p in GBM tissues.

HSV1 miR	Target	Log_2_FC	Adjusted P-value
hsv1-miR-H6-3p	*SH3TC2*	− 3.64	0.006
	*SASH1*	− 1.39	0.022
	*EPB41L1*	− 1.51	0.022
	*SH3PXD2A*	− 1.98	0.039
hsv1-miR-H1-5p	*MELK*	4.285	0.004
	*FZD2*	2.186	0.008
	*IL1RN*	2.178	0.012
	*NOVA1*	1.622	0.013
	*TMEM97*	1.489	0.022
	*PTPRZ1*	1.48	0.029
	*PDGFC*	1.39	0.045

*MYH10* is not included in this table because external validation has not validated its upregulation in GBM tissues.

### Characterizing hsv1-miR-H6-3p targets

**Figure 3:**
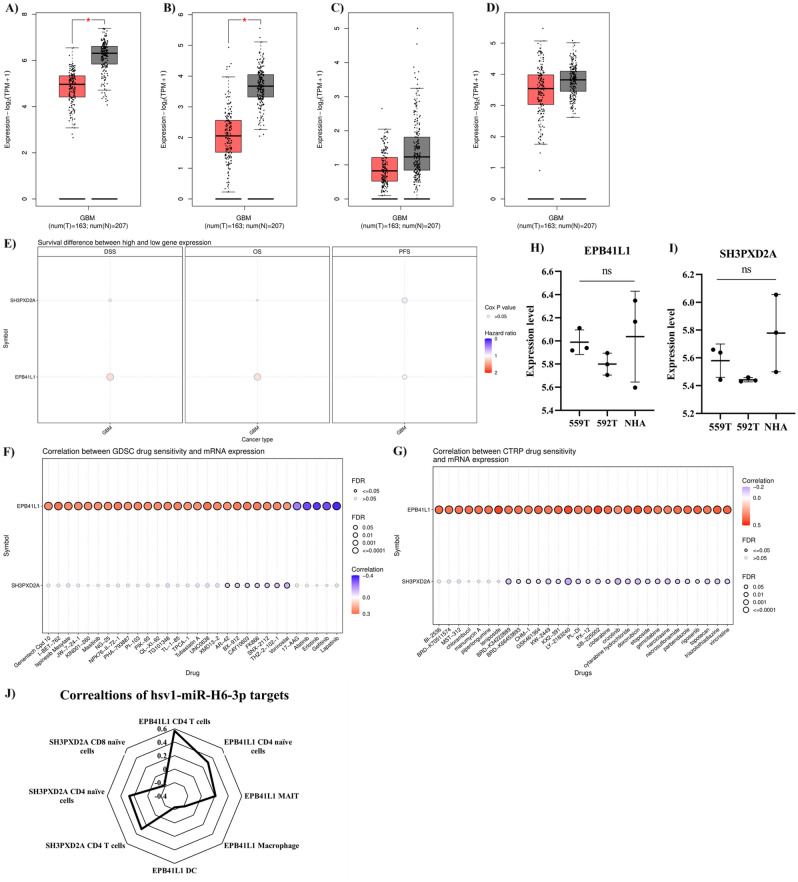
Characterizing hsv1-miR-H6-3p targets. (**A**) *EPB41L1* expression, (**B**) *SH3PXD2A* expression, (**C**) *SH3TC2* expression, (**D**) *SASH1* expression, (**E**) the prognostic values of *EPB41L1* and *SH3PXD2A* in GBM patients. (**F**) Drug sensitivity analysis of *EPB41L1* and *SH3PXD2A* based on GDSC. (**G**) Drug sensitivity analysis of *EPB41L1* and *SH3PXD2A* based on CTRP. (**H**,**I**) *EPB41L1* and *SH3PXD2A* expression in temozolomide-resistant GBM cell lines and normal astrocyte cells. (**J**) Immune cell infiltration and *EPB41L1* and *SH3PXD2A* in GBM tissues.

We accessed the TCGA-GBM and GTEX datasets to study the expression levels of the identified gene. We used GBM tissues (*n* = 163) from the TCGA-GBM dataset and normal brain tissues (*n* = 207) from the GTEX dataset. Our results have confirmed that the expression levels of *EPB41L1* and *SH3PXD2A* are significantly downregulated in GBM tissues (Fig. 3A,B). However, there are no statistically significant differences in the expression levels of *SH3TC2* and *SASH1* between GBM and non-tumoral tissues (Fig. 3C,D). Based on this external validation, hsv1-miR-H6-3p upregulation can be responsible for downregulating *EPB41L1* and *SH3PXD2A* expression in GBM tissues.

After externally validating *EPB41L1* and *SH3PXD2A* downregulation in GBM, we studied their prognostic values. It has been found that *EPB41L1* and *SH3PXD2A* have no statistically significant prognostic values in determining the disease-specific survival, overall survival, and progression-free survival of GBM patients (Fig. 3E). Figure 3F,G depict the drug sensitivity analyses. Of note, there is a significant positive correlation between *EPB41L1* with teniposide, as anti-neoplastic chemotherapy. However, no significant correlations were identified between *EPB41L1* and *SH3PXD2A* with temozolomide. Consistent with this, it has been found that there are no significant differences between the expression levels of *EPB41L1* and *SH3PXD2A* in temozolomide-resistant cells and normal astrocyte cells (Fig. 3H,I). Figure 3J shows the correlations between *EPB41L1* and *SH3PXD2A* and infiltrated immune cells in GBM tissues; of note, there is a significant positive correlation between CD4^+^ T-cells and *EPB41L1* in GBM tissues. The enrichment analysis has indicated that *SH3PXD2A* is significantly involved in the superoxide-generating NADPH oxidase activator activity (data are not shown).

### Characterizing hsv1-miR-H1-5p targets

**Figure 4:**
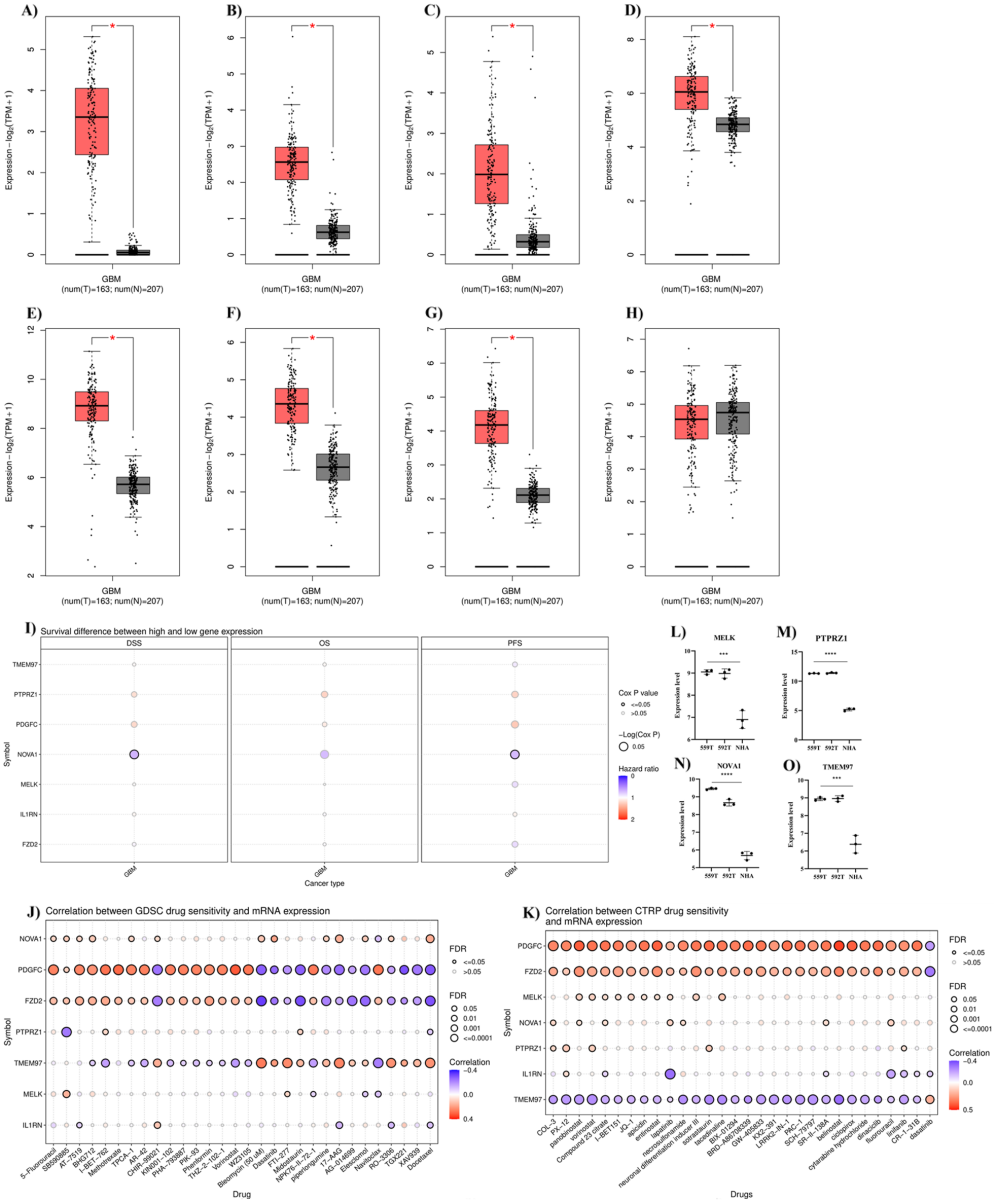

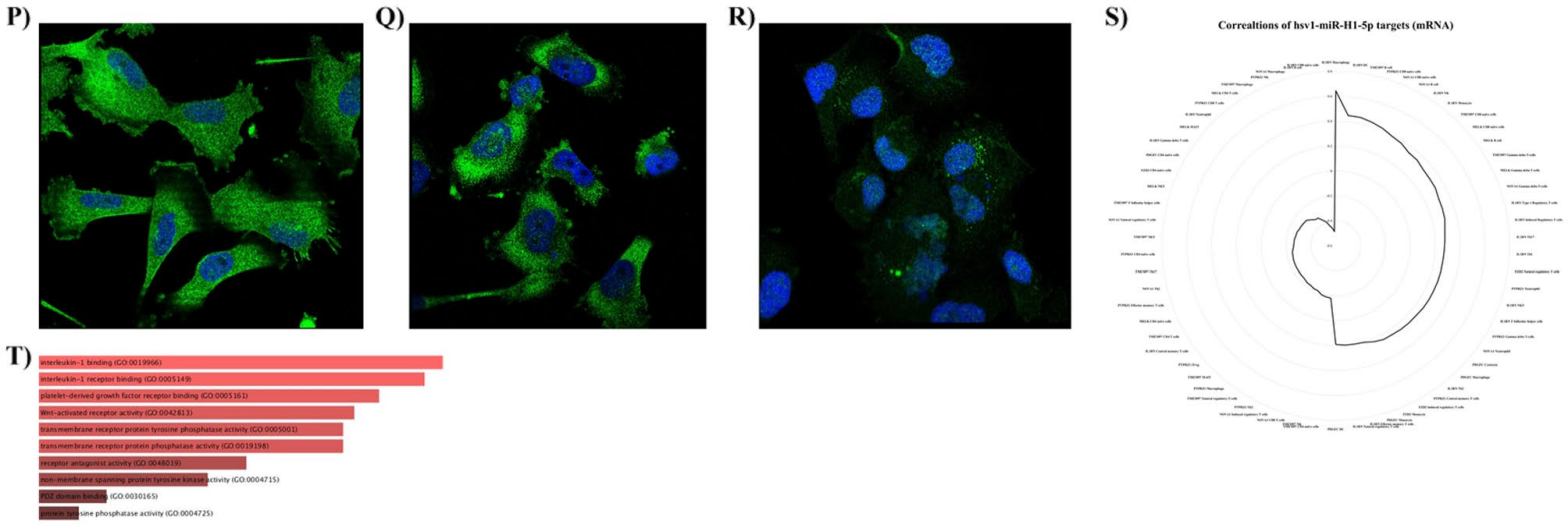
Characterizing hsv1-miR-H1-5p targets. (**A**) *MELK* expression, (**B**) *FZD2* expression, (**C**) *IL1RN* expression, (**D**) *NOVA1* expression, (**E**) *PTPRZ1* expression, (**F**) *TMEM97* expression, (**G**) *PDGFC* expression, (**H**) *MYH10* expression, (**I**) the prognostic values of *MELK, FZD2, IL1RN, NOVA1, PTPRZ1, TMEM97, *and* PDGFC* in GBM patients. (**J**) Drug sensitivity analysis of *MELK, FZD2, IL1RN, NOVA1, PTPRZ1, TMEM97*, and *PDGFC* based on GDSC. (**K**) Drug sensitivity analysis of *MELK, FZD2, IL1RN, NOVA1, PTPRZ1, TMEM97, and PDGFC* based on CTRP. (**L**–**O**) *MELK, PTPRZ1, NOVA1,* and *TMEM97* expression in temozolomide-resistant GBM cell lines and normal astrocyte cells. (**P**) PDGFC staining in GBM cells, (**Q**) PTPRZ1 staining in GBM cells, (**R**) NOVA1 staining in GBM cells. (**S**) Immune cell infiltration and hsv1-miR-H1-5p targets in GBM tissues. (**T**) Enrichment analyses of hsv1-miR-H1-5p targets.

For the external validation, we investigated the expression levels of identified hsv1-miR-H1-5p targets based on TCGA-GTEX data. The expression levels of *MELK*, *FZD2*, *IL1RN*, *NOVA1*, *PTPRZ1*, *TMEM97*, and *PDGFC* are significantly upregulated in GBM tissues (Fig. 4A–G). However, *MYH10* expression is not altered in GBM tissues (Fig. 4H). Based on this external validation, hsv1-miR-H1-5p downregulation can be responsible for upregulating *MELK*, *FZD2*, *IL1RN*, *NOVA1*, *PTPRZ1*, *TMEM97*, and *PDGFC* in GBM tissues.

**Figure 5:**
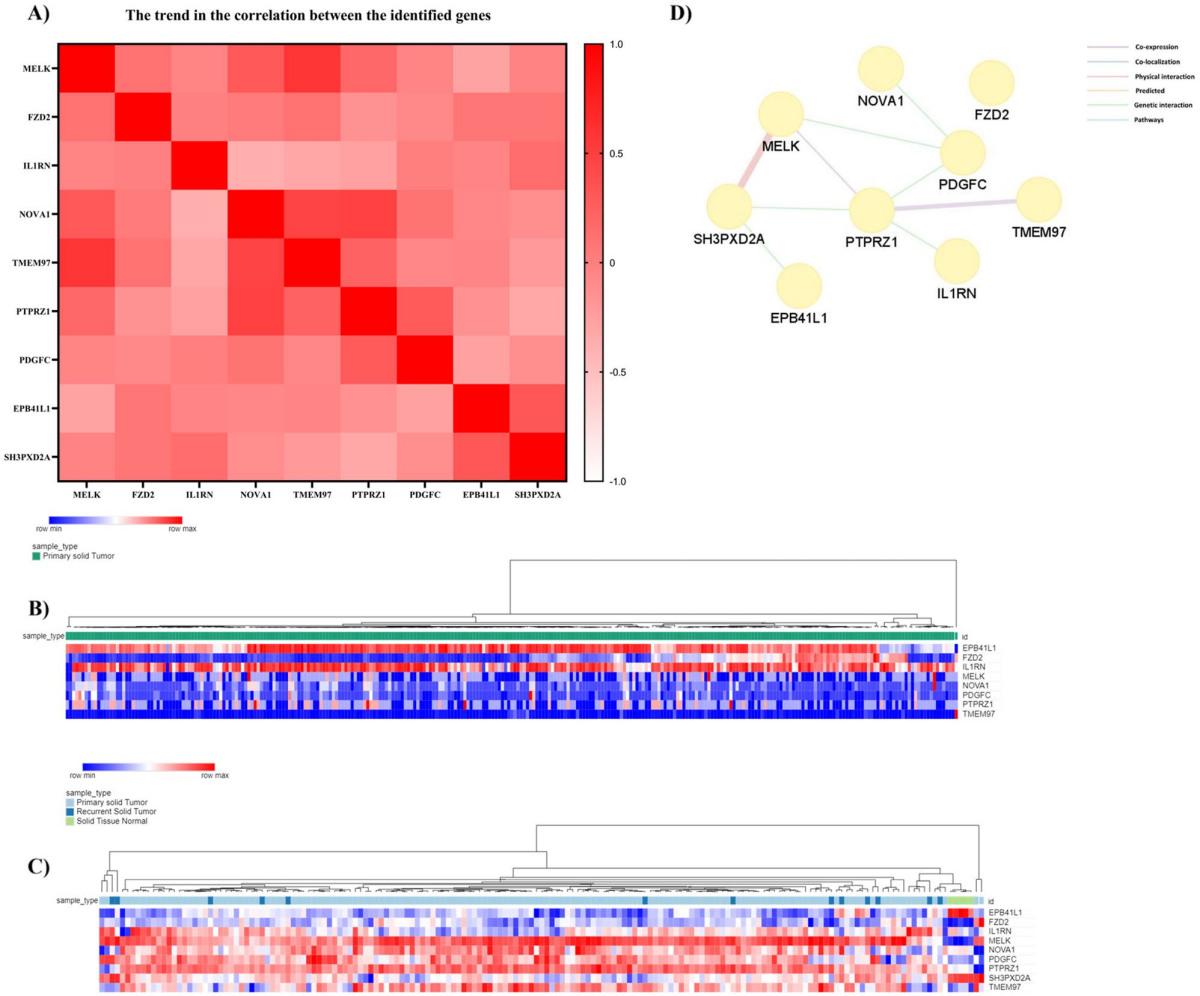
Tumor bulk analyses and gene interactions. (**A**) The heatmap of the correlation between the identified common genes based on the TCGA-GBM dataset. (**B**) The heatmap of methylation patterns of hsv1-miR-H6-3p and hsv1-miR-H1-5p targets. (**C**) The heatmap of mRNA expression patterns of hsv1-miR-H6-3p and hsv1-miR-H1-5p targets. (**D**) The gene interaction of hsv1-miR-H6-3p and hsv1-miR-H1-5p targets based on GeneMANIA.

Following the external validation of *MELK*, *FZD2*, *IL1RN*, *NOVA1*, *PTPRZ1*, *TMEM97*, and *PDGFC* upregulation in GBM, we studied their prognostic significance. It has been found that *NOVA1* downregulation is significantly associated with poor disease-specific survival and progression-free survival of GBM patients (Fig. 4I). Figure 4J,K depict the drug sensitivity analyses. Notably, there are significant negative correlations between the studied upregulated genes with bleomycin, midostaurin, afatinib, and docetaxel, as anti-neoplastic chemotherapeutic agents. Only the expression of *TMEM97* has been positively associated with temozolomide treatment based on GDSC. However, the expression levels of *MELK*, *PTPRZ1*, and *NOVA1,* along with *TMEM97,* have been significantly upregulated in temozolomide-resistant GBM cell lines compared to normal astrocyte cells (Fig. 4I–O). The HPA009134, HPA015103, and HPA004155 staining have localized the PDGFC in the plasma membrane and cytosol, PTPRZ1 in the cytosol, and NOVA1 in the nucleoplasm, nucleoli, and vesicles, respectively (Fig. 4P–R, respectively). Figure 5S shows the correlations between *MELK*, *FZD2*, *IL1RN*, *NOVA1*, *PTPRZ1*, *TMEM97*, and *PDGFC* and infiltrated immune cells in GBM tissues; of note, there is a significant positive correlation between macrophage and *IL1RN* and negative correlation between CD8^+^ naïve T-cells and *IL1RN* in GBM tissues. The enrichment analysis has indicated that *FZD2* and *PTPRZ1* are significantly involved in Wnt-activated receptor activity and protein tyrosine phosphatase activity, respectively (Fig. 4T).

### Correlational studies and gene interaction of hsv1-miR-H6-3p and hsv1-miR-H1-5p targets

The data from the TCGA-GBM were analyzed to demonstrate a trend in the correlation of the identified genes (Fig. 5A). The TCGA-GBM data has releveled an overall hypomethylation in *MELK*, *FZD2*, *IL1RN*, *NOVA1*, *PTPRZ1*, *TMEM97*, and *PDGFC* and hypermethylation in *EPB41L1* in GBM tissues (Fig. 5B). Figure 5C depicts the mRNA expression patterns of hsv1-miR-H6-3p and hsv1-miR-H1-5p in GBM and non-tumoral tissues. Except for *FZD2*, hsv1-miR-H6-3p and hsv1-miR-H1-5p targets have considerable interactions with each other based on the GeneMANIA (Fig. 5D).

### The expression pattern of identified common genes in cell populations of GBM tissues

**Figure 6:**
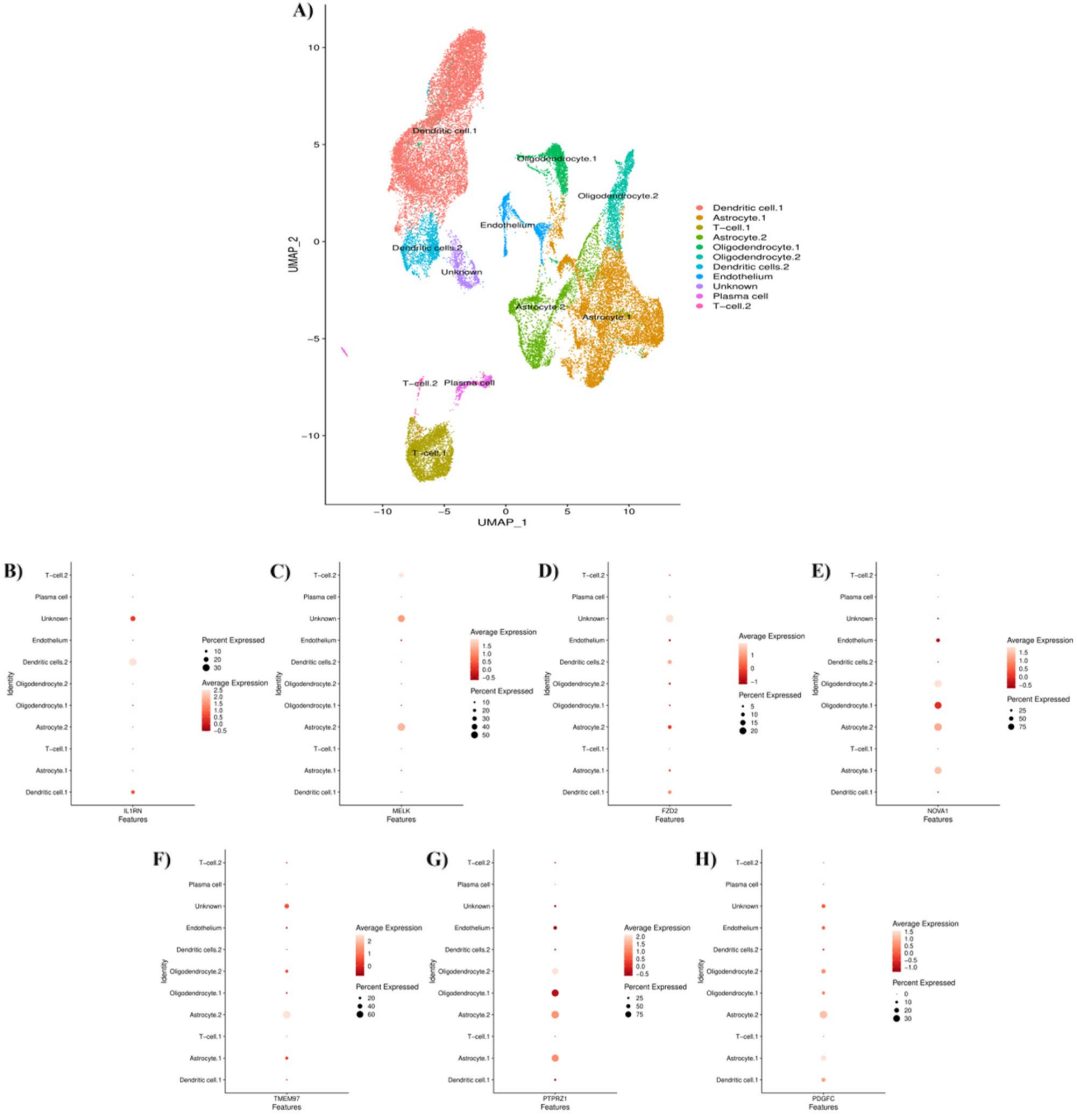
Identification of cells in the included primary GBM microenvironment and the dot plots of the identified genes for demonstrating their expression patterns in the cell populations of the included primary GBM microenvironment. (**A**) Cell populations of included GBM tissues, (**B**) *IL1RN*, (**C**) *MELK*, (**D**) *FZD2*, (**E**) *NOVA1*, (**F**) *TMEM97*, (**G**) *PTPRZ1*, and (**H**) *PDGFC.*

After external validating the upregulation of *MELK*, *FZD2*, *IL1RN*, *NOVA1*, *TMEM97*, *PTPRZ1*, and *PDGFC* in GBM tissues compared to non-tumoral samples, we applied single-cell RNA sequencing analyses to identify which cell types are responsible for the increased expression of these genes. After quality control, 35,133 out of 42,966 cells were included in our analyses. Our results have identified 11 distinct cell populations in the included primary GBM tissues (Fig. 6A). Except for the *IL1RN*, *MELK*, *FZD2*, *NOVA1*, *TMEM97*, *PTPRZ1*, and *PDGFC* have been highly expressed in astrocyte populations in terms of magnitude and/or intensity (Fig. 6B–H, respectively).

## Discussion

Despite considerable advancements in our understanding of GBM development, the underlying virus-mediated post-transcriptional regulation in GBM development needs further studies. In the current in silico study, we thoroughly investigated the role of HSV1 miRs in GBM development. We identified that hsv1-miR-H6-3p, as an upregulated HSV1 miR in GBM tissues, can target the expression of *EPB41L1* and *SH3PXD2A* in GBM tissues. Also, hsv1-miR-H1-5p, as a downregulated HSV1 miR in GBM tissues, can target the expression of *MELK*, *FZD2*, *NOVA1*, *TMEM97, PTPRZ1*, and *PDGFC* in non-tumoral brain tissues and these genes are expressed in astrocytes. The following discusses the significance of the above-mentioned genes in GBM development.

As a cell-cycle-dependent protein kinase overexpressed in cancer cells, *MELK* silencing is associated with an increased level of *p21* in *p53*-deficient cells and arrests cancer cells at the G1 phase^14^. *MELK* inhibitor has been associated with enhanced radiosensitivity and suppressed tumor growth in animal models^15^. *MELK* inhibitor suppresses the proliferation, clonogenicity, neurosphere formation, invasion, and migration of GBM cells. *MELK* inhibition can also upregulate *p21* and arrest the cell cycle. In animal models, *MELK* inhibition has increased the survival of affected mice and inhibited tumor growth. Of interest, the inhibitory effect of *MELK* inhibition has been more effective on cancer stem cells than on GBM cells^16^. *FZD2* can stimulate the canonical and non-canonical Wnt pathways in malignancies. *FZD2* suppression inhibits the β-catenin-dependent signaling pathway, and its knockdown suppresses tumor growth in neuroblastoma animal models^17^. *FZD2* meditates the EMT process and cell migration, and administering antibodies against *FZD2* suppresses tumor growth and migration in animal models^18^. In line with these, we have shown that *FZD2* is significantly involved in the Wnt-activated receptor activity. *NOVA1* can increase telomerase activity and enhances its stability, leading to tumor development^19,20^. *NOVA1* is substantially upregulated in GBM tissues and GBM cells compared to non-tumoral counterparts. *NOVA1* expression is upregulated in patients with high-infiltration depth and tumors greater than 2 cm compared with patients with low infiltration depth and smaller tumor sizes, respectively^21^. *TMEM97* expression is substantially increased in glioma tissues compared to non-tumoral brain tissues, and its expression is increased with higher glioma grades. Increased expression of *TMEM97* has also been associated with the inferior survival of glioma patients. *TMEM97* silencing suppresses tumor proliferation, arrests the cell cycle, inhibits cell invasion, and decreases the migration of GBM cells. Besides, *TMEM97* knockdown suppresses the EMT process, decreases the protein expression of β-catenin and Twist, and downregulates E-cadherin protein expression in GBM cells^22^. *TMEM97 *knockdown can suppress astrocytoma migration and decrease tumor growth^22^. *PTPRZ1* expression has been higher in glioma stem cells compared to matched non-tumoral stem cells, and *PTPRZ1* knockdown decreases the cell viability and tumorsphere formation of glioma stem cells. In animal models, *PTPRZ1* disruption decreases cell proliferation and downregulates SOX2 expression. Besides, *PTPRZ1*^+^ tumor cells display a high rate of tumorigenesis compared with matched *PTPRZ1*^-^ in animal models^23^. Fujikawa et al. have shown that *PTPRZ1* knockdown decreases stemness features of GBM cells^24^. Activating the platelet-derived growth factor receptor (PDGFR) pathway via PDGFs can stimulate oncogenic effects and lead to GBM development. *PDGFC* expression is substantially higher in GBM tissues than in normal brain tissues. Also, *PDGFC* expression is upregulated in GBM cells and GBM stem cells compared to normal astrocyte cells^25^. Besides, *PDGFC* knockdown remarkably decreases non-small cell lung cancer cell proliferation^26^. The increased expression of *PDGFC* is associated with the inferior disease-free survival of patients with triple-negative breast cancers^27^. Besides, upregulated *PDGFC* expression is associated with the histological grade and stage of breast cancer patients, and its knockdown has decreased invasion and proliferation in breast cancer cells^39^.

The mRNA and protein expression of *EPB41L1* is considerably downregulated in GBM tissues compared to adjacent non-tumoral tissues. *EPB41L1* overexpression arrests the cell cycle in the G0/G1 phase, increases the apoptosis rate, and suppresses the invasion and migration of GBM cells^28^. Our results have demonstrated that *EPB41L1* is considerably downregulated in GBM tissues, and hsv1-miR-H6-3p might target their expression. We have demonstrated that *SH3PXD2A* is significantly involved in the superoxide-generating NADPH oxidase activator activity. It is found that the expression level of *SH3PXD2A* is substantially decreased in GBM tissues, and hsv1-miR-H6-3p might target their expression.

The current study has some strengths. First, we highlighted the trace of HSV1 miRs in GBM development. Second, we applied exhaustive in silico investigations, external validation, and literature review to elucidate the identified HSV1 miRs targets in GBM pathogenesis. Third, we applied single-cell RNA analyses to display the expression patterns of the upregulated genes that can be targeted by hsv1-miR-H1-5p in various cells residing in the GBM microenvironment. Overall, the current study provides novel insights into the potential role of HSV1 miRs in GBM development.

## Methods

### Dataset selection

The Gene Expression Omnibus (GEO) database of NCBI was deeply searched to select datasets that investigated miR expression in GBM and non-tumoral tissues. For this purpose, the GSE158284 database was selected; this dataset utilized Agilent-021827 Human miRNA Microarray (V3) (Probe Name version) to investigate miR expression in GBM and non-tumoral tissues. Besides, we aimed to identify datasets that studied mRNA expression in GBM tissues, adjacent non-tumoral tissues, GBM cell lines, and normal astrocyte cells. For this purpose, the GSE13276 dataset was chosen; this dataset used [HG-U133A] Affymetrix Human Genome U133A Array to study mRNA expression in GBM and non-tumoral samples^29^. Also, we selected the GSE153679 that sequenced the gene expression of normal astrocyte cells and patient-derived temozolomide-resistant GBM cell lines. This dataset applied [HuGene-1_0-st] Affymetrix Human Gene 1.0 ST Array [transcript (gene) version] for sequencing genes^30^. For single-cell RNA sequencing, the GSE182109 dataset was selected to access the raw single-cell RNA sequencing data of patients with primary GBM. The GSE182109 dataset applied Illumina HiSeq 4000 (*Homo sapiens*) for single-cell RNA sequencing^31^.

### Identifying differentially expressed genes (DEGs) and HSV1 miRs targets

After data normalization and clustering, R software (version 4.1.3) was leveraged to find the differentially expressed miRs and mRNAs in GSE158284 and GSE13276. Like our previous work, the Benjamini and Hochberg method was used for adjusting the obtained P-values^2^. The *limma* package was used to identify DEGs. The adjusted P-value ˂ 0.05 and |log2FC| ≥ 1.3 were the criteria for these analyses considering a miR or an mRNA as significantly altered miR or mRNA expression. Volcano plots were drawn to display the differentially expressed miRs and mRNAs. After identifying the differentially expressed HSV1 miRs, the miRDB database was used to identify the potential targets of HSV1 miRs^32^. A score ≥ 50 was the criteria for selecting the targets of HSV1 miRs.

### Tumor bulk analysis

The TCGA-GBM and GTEX datasets were accessed using the GEPIA2 (http://gepia2.cancer-pku.cn/#index) to validate the expression of identified DEGs externally^33^. The TCGA-GBM dataset was also analyzed to find the correlations between DEGs, their mRNA expression, and methylation patterns in the non-tumoral and GBM tissues. The survival, immunogenetics, and drug sensitivity analyses were conducted using gene set cancer analysis (GSCA). This database contains data from genomics of drug sensitivity in cancer (GDSC), cancer therapeutics response portal (CTRP), and TCGA cohorts. The immunogenetics analyses are based on the immuCellAI algorithms^34^. The Human Protein Atlas (https://www.proteinatlas.org/) was used to identify the protein localization of DEGs in human GBM cell lines^35^. The molecular function of DEGs was investigated using enrichment analyses. Adjusted P-value < 0.05 was considered a criterion for determining the significance^36^.

### Single-cell RNA sequencing analyses

The GSE182109 dataset was downloaded from the Gene Expression Omnibus (GEO) database (https://www.ncbi.nlm.nih.gov/geo/). The *Seurat* (version 4.1.0)^37^ R package was used to analyze raw single-cell RNA sequencing data. Cells with mitochondrial gene expression ˂ 10% and expressed genes of above 500 were selected for analysis. Following data normalization, finding variable genes, and integrating data from multiple samples, we scaled the data and ran the principal component analysis. Afterward, the cells were clustered into a two-dimensional figure using the uniform manifold approximation and projection (UMAP) clustering algorithms with a resolution of 0.1. The PanglaoDB^38^ and CellMarker^39^ databases were used to annotate the identified clusters. The gene markers used in this study are shown in Table 2.

**Table 2:** The gene markers used for annotating the identified clusters.

Cell population	Gene markers
Dendritic cell.1	*HLA-DRA, HLA-DPA1, HLA-DPB1, FCGR3A, HLA-DRB1, HLA-DQA1, HLA-DMA, HLA-DMB, CXCL8, LYZ, PTPRC, ITGAM,* and *CD68*
Astrocyte.1	*CLU, GFAP, AQP4, C1orf61, CRYAB, SPARCL1, ID4, CPE, SOX2, OLIG1, GFAP,* and *S100B*
T-cell.1	*IL32, GZMH, PYHIN1, CD8B, PTPRC, CD3E,* and *CD8A*
Astrocyte.2	*AGT, ANLN, ARHGEF26, ATP13A4, ATP1B2,* and *C1orf61*
Oligodendrocyte.1	*MBP, PLP1, CLDN11, MAG, PPP1R14A, PLEKHB1, SIRT2, CNP,* and* TF*
Oligodendrocyte.2	*ERMN, UGT8, ZCCHC24, GPR37, MAL, FERMT1, SERINC5, OMG, PHACTR3, NEU4, AMOTL2, TMEFF2, MYT1, DLL1, MAP6D1, ANGPTL2,* and *SNX1*
Dendritic cells.2	*S100A9, S100A8, EREG, SLC2A3, FCN1, CD36, ANPEP, ADAM8, THBD, TREM1, CD300E, FGR, CHST15, RXRA, ASGR1, ATF5, CTSL1, ITGA5, FLNB, GNS,* and *HK1*
Endothelium	*PECAM1, ADIRF1, A2M, APOLD1, FLT1, CCL14,* and *PCAT19*
Plasma cell	*FCRL2, IGHG2, IGHGP, IGHA1, IGHG4,* and *IGLV3-1*
T-cell.2	*IL32, GZMH, CCL4L2, PYHIN1,* and* CD3E*

## Data Availability

The GSE158284, GSE13276, GSE153679, and GSE182109 datasets were downloaded from GEO (https://www.ncbi.nlm.nih.gov/geo/). The TCGA-GBM and GTEX datasets were accessed using the GEPIA2 (http://gepia2.cancer-pku.cn/#index). The Human Protein Atlas (https://www.proteinatlas.org/) was acessed for protein localization.

## References

[1] Miller KD (2021). Brain and other central nervous system tumor statistics, 2021. CA Cancer J. Clin..

[2] Shadbad MA (2021). A Systematic review on the therapeutic potentiality of PD-L1-inhibiting microRNAs for triple-negative breast cancer: Toward single-cell sequencing-guided biomimetic delivery. Genes.

[3] Shadbad MA (2021). A scoping review on the potentiality of PD-L1-inhibiting microRNAs in treating colorectal cancer: Toward single-cell sequencing-guided biocompatible-based delivery. Biomed. Pharmacother..

[4] Rezaei T (2020). microRNA-181a mediates the chemo-sensitivity of glioblastoma to carmustine and regulates cell proliferation, migration, and apoptosis. Eur. J. Pharmacol..

[5] Dastmalchi N (2021). An updated review of the cross-talk between microRNAs and epigenetic factors in cancers. Curr. Med. Chem..

[6] Buruiană A (2020). The roles of miRNA in glioblastoma tumor cell communication: Diplomatic and aggressive negotiations. Int. J. Mol. Sci..

[7] Naqvi AR, Shango J, Seal A, Shukla D, Nares S (2018). Herpesviruses and microRNAs: New pathogenesis factors in oral infection and disease?. Front. Immunol..

[8] Lei T (2013). Targeting of DICE1 tumor suppressor by Epstein–Barr virus-encoded miR-BART3* microRNA in nasopharyngeal carcinoma. Int. J. Cancer.

[9] Wong AMG, Kong KL, Tsang JWH, Kwong DLW, Guan XY (2012). Profiling of Epstein–Barr virus-encoded microRNAs in nasopharyngeal carcinoma reveals potential biomarkers and oncomirs. Cancer.

[10] Starr JR (2001). Serologic evidence of herpes simplex virus 1 infection and oropharyngeal cancer risk. Cancer Res..

[11] Jalouli J (2012). Human papilloma virus, herpes simplex virus and Epstein Barr virus in oral squamous cell carcinoma from eight different countries. Anticancer Res..

[12] Brown SH, Afghan AK, Satyanarayana G (2022). Herpes simplex virus-infected squamous cell carcinoma: A case report. BMC Infect. Dis..

[13] Jasim A, Proietto A, Scurry J (2016). Herpes simplex virus infection of vulvar squamous cell carcinoma. Pathology.

[14] Matsuda T (2017). p53-independent p21 induction by MELK inhibition. Oncotarget.

[15] Speers C (2016). Maternal embryonic leucine zipper kinase (MELK) as a novel mediator and biomarker of radioresistance in human breast cancer. Clin. Cancer Res..

[16] Zhang X (2021). MELK inhibition effectively suppresses growth of glioblastoma and cancer stem-like cells by blocking AKT and FOXM1 pathways. Front. Oncol..

[17] Zins K (2016). Frizzled2 signaling regulates growth of high-risk neuroblastomas by interfering with β-catenin-dependent and β-catenin-independent signaling pathways. Oncotarget.

[18] Gujral TS (2014). A noncanonical Frizzled2 pathway regulates epithelial-mesenchymal transition and metastasis. Cell.

[19] Sayed ME (2019). NOVA1 directs PTBP1 to hTERT pre-mRNA and promotes telomerase activity in cancer cells. Oncogene.

[20] Ludlow AT (2018). NOVA1 regulates hTERT splicing and cell growth in non-small cell lung cancer. Nat. Commun..

[21] Jin L (2019). MicroRNA-193a-5p exerts a tumor suppressor role in glioblastoma via modulating NOVA1. J. Cell. Biochem..

[22] Qiu G (2015). RNA interference against TMEM97 inhibits cell proliferation, migration, and invasion in glioma cells. Tumor Biol..

[23] Shi Y (2017). Tumour-associated macrophages secrete pleiotrophin to promote PTPRZ1 signalling in glioblastoma stem cells for tumour growth. Nat. Commun..

[24] Fujikawa A (2017). Targeting PTPRZ inhibits stem cell-like properties and tumorigenicity in glioblastoma cells. Sci. Rep..

[25] Yang Y (2019). MicroRNA-29a inhibits glioblastoma stem cells and tumor growth by regulating the PDGF pathway. J. Neuro-oncol..

[26] McDermott U (2009). Ligand-dependent platelet-derived growth factor receptor (PDGFR)-alpha activation sensitizes rare lung cancer and sarcoma cells to PDGFR kinase inhibitors. Cancer Res..

[27] Kim S (2021). Inhibition of platelet-derived growth factor C and their receptors additionally increases doxorubicin effects in triple-negative breast cancer cells. Eur. J. Pharmacol..

[28] Han X, Wang X, Li H, Zhang H (2019). Mechanism of microRNA-431-5p-EPB41L1 interaction in glioblastoma multiforme cells. Arch. Med. Sci. AMS.

[29] Mangiola A (2013). Gene expression profile of glioblastoma peritumoral tissue: An ex vivo study. PLoS One.

[30] Nam HJ (2021). Azathioprine antagonizes aberrantly elevated lipid metabolism and induces apoptosis in glioblastoma. iScience.

[31] Abdelfattah N (2022). Single-cell analysis of human glioma and immune cells identifies S100A4 as an immunotherapy target. Nat. Commun..

[32] Chen Y, Wang X (2020). miRDB: An online database for prediction of functional microRNA targets. Nucleic Acids Res..

[33] Tang Z, Kang B, Li C, Chen T, Zhang Z (2019). GEPIA2: An enhanced web server for large-scale expression profiling and interactive analysis. Nucleic Acids Res..

[34] Liu CJ (2023). GSCA: An integrated platform for gene set cancer analysis at genomic, pharmacogenomic and immunogenomic levels. Brief. Bioinform..

[35] Sjöstedt E (2020). An atlas of the protein-coding genes in the human, pig, and mouse brain. Science.

[36] Xie Z (2021). Gene set knowledge discovery with Enrichr. Curr. Protoc..

[37] Hao Y (2021). Integrated analysis of multimodal single-cell data. Cell.

[38] Franzén O, Gan L-M, Björkegren JL (2019). PanglaoDB: A web server for exploration of mouse and human single-cell RNA sequencing data. Database.

[39] Zhang X (2019). Cell Marker: A manually curated resource of cell markers in human and mouse. Nucleic Acids Res..

